# International accounting network memberships and audit fees: Evidence from China

**DOI:** 10.1371/journal.pone.0296304

**Published:** 2024-01-18

**Authors:** Xuemei Xiong, Ruoting Zheng, YanJian Liu, Xuanhao Huang

**Affiliations:** 1 Newhuadu Business School, Minjiang University, Fuzhou, China; 2 School of Economics and Management, Fuzhou University, Fuzhou, China; Privatuniversität Schloss Seeburg: Privatuniversitat Schloss Seeburg, AUSTRIA

## Abstract

This paper investigates the international accounting network memberships’ impact on audit fees. We find that, firstly, the audit fees charged by the member audit firms are significantly higher; secondly, if the revenue, ranking or audit and accounting business share of the international network the audit firm join is higher, the charge is also higher. Additional results show that economic policy uncertainty will intensify this positive relationship, and member audit firms charge higher fees by improving their overseas expertise. We also find that international network memberships will reduce abnormal audit fees, and improve the quality of financial reports.

## 1. Introduction

Accelerated globalization has led to frequent cross-border trade, and audit firms, as important intermediaries in the capital market, are inevitably caught up in the wave of internationalization. In 2007, CICPA issued the "Opinions on Promoting Accounting Firms to Grow and Strengthen", which clearly pointed out that establish or join international networks is an important way for the internationalization of audit firms. In 2009, the Ministry of Finance issued the "Opinions on Accelerating the Development of China’s Certified Public Accountant Industry" to vigorously support audit firms to grow bigger and stronger. Audit firms that join international networks (for brevity, international accounting associations, networks, or alliances are referred to as networks) can share the technology, reputation, customers and other resources of them, but at the same time, they also need to pay membership fees and accept the quality inspection or other agreements of the international networks. Most of the current studies generally conclude that joining an international network improves audit quality [1]. By joining an international network, the audit firm can obtain richer audit resources, but it also will be subject to more stringent regulatory standards that may change the auditor’s expected input and audit risk, which in turn affects audit fees. Therefore, we are acutely aware of the question that is the audit service provided by the members of international networks "high quality and inexpensive" or "high quality and high price"?

Audit fees are the audit client’s reward for the auditor’s work, which not only reflect the auditor’s input, but also embody the auditor’s insurance capability. By joining an international network, the audit firm has an access to a wider range of professional and technical support, and the collaboration and communication among network members can improve audit efficiency, and this knowledge spillover effect leads to lower audit costs and hence lower audit fees. On the other hand, when negotiating the price of audit services with clients, network members may demand higher audit fees because of their reputation and brand value, or compensation for costs due to membership fees to international networks. So, it is worth studying whether international network members charge higher fees than non-members. Further, with respect to the size, ranking and expertise of the networks, will these characteristics affect audit fees? And we also try to find channels through which network members influence audit fees.

To address these concerns, we use Chinese A-share listed companies from 2007–2019 as the research sample. The empirical results show that, first, network members charge significantly higher audit fees compared to non-members; and second, if the network that the audit firm joined has a higher revenue, ranking, or proportion of audit and accounting business, audit fees are significantly higher. These results imply that network members are more inclined to charge higher fees because of higher quality services, and the specific characteristics of the international accounting networks that the audit firms joined also have a significant impact on audit fees. Further analyses show that network members affect audit fees through the path of increasing overseas expertise by using intermediary effect model, and economic policy uncertainty exacerbates the positive relationship between audit firms’ network memberships and audit fees; moreover, international network memberships are negatively associated with abnormal audit fees and discretional accruals. These findings indicate that higher audit fees charged by network members are more likely to be additional compensation for auditors’ hard work to improve financial reporting quality rather than a risk compensation for audit collusion.

Our findings contribute to prior literature in several ways. First, existing researches on the international accounting networks have focused only on whether audit firms join international networks but ignored their specific characteristics [1–3], this paper further investigate the specific characteristics, such as revenue, ranking, and expertise of the international accounting networks, which can deepen the researches on the relationship between member firms and audit fees. Second, prior studies have not paid much attention to the specific mechanism through which the internationalization of accounting firms affect audit fees, our study try to bridge the gap and find that network members increase audit fees through the path of increasing auditors’ overseas expertise; meanwhile, in the test of economic consequences, we find that despite the increase in audit fees, international network memberships have not lead to an increase in abnormal audit fees (usually used as a proxy variable for audit opinion purchase) and have also improved the quality of financial reporting, suggesting that higher audit fees are charged due to extra audit efforts rather than collusion, these findings complement prior studies and try to give a reasonable explanation for higher fees charged by member firms. Third, this paper attempts to further understand the impact of Chinese local audit firms’ international network memberships, Mao et al. (2017) find that member firms charge higher audit fees but make no significant improvement in audit quality, they attribute this to China’s weak institutional environment which may offset the benefits of joining networks [2]. However, our study finds that firms joining international networks not only increase audit fees but also improve financial reporting quality, which is different from the conclusion of Mao et al. [2].

The remainder of this paper is organized as follows. In Section 2, we review the relevant literature and develop our main hypotheses. Section 3 introduces the research design, including sample selection and data sources, variable definition and empirical model. Section 4 reports the empirical results. Section 5 conducts some further analyses, including cross-sectional analysis, the intermediary effect test and economic consequences test. Section 6 presents our robustness tests. Finally, Section 7 provides conclusions and recommendations.

## 2. Literature review and hypothesis development

### 2.1 Literature review

Existing studies on the influence of international accounting networks’ memberships have mostly focused on audit quality, while the findings are not uniform in different settings. Using US companies as the research examples, Bills et al. (2016) found that audit firms that joined international networks had higher audit quality and higher audit fees [1]. However, using data of Chinese listed companies from 2001 to 2010, Mao et al. (2017) found that member audit firms charge higher audit fees but make no significant improvement in audit quality [2]. By surveying 37 partners in 18 U.S. accounting firms from nine different international networks, Bills et al. (2018) found that smaller firms often use international network memberships to obtain necessary resources and improve their market position, and that most respondents believe that the resources provided by international networks, especially expertise, are important for audit quality improvement [3]. Saito and Takeda (2014) used the audit scandal at PricewaterhouseCoopers in Chuo Aoyama, Japan, as a study case and found that audit failures by network members negatively affect the reputation of other members, with a significant drop in the price of their audit client’s securities, suggesting that the reputation of all members is tied together [4]. Carson (2009) argued that the development of network alliances can facilitate the formation of global industry specializations in accounting firms and consequently increase audit fee premiums [5].

Simunic (1980) was the first to examine the influencing factors of audit fees using regression models, and the study found that asset size, the number of subsidiaries and industries involved, gearing ratio, profitability, and the audit opinion significantly affect audit fees [6]. In general, existing research on the factors affecting audit fees can be categorized into two aspects, namely, audit client specifics and auditor specifics. First, with respect to audit clients, earlier studies found that company size, business complexity, financial position, and profitability etc. could affect audit fees [7–9]. Auditors generally need to spend more time to complete the audit engagement when the company is larger in size or the company’s activities are more complex [10, 11], and therefore the audit fee will also be higher; better profitability or financial position typically implies lower risk of the client, thus leading to lower audit fees. In addition, some scholars have also focused on the effects of earnings management, audit committee characteristics, internal control, etc. on audit fees [12–15], these factors can be categorized as corporate governance. Recent studies have further refined audit client specifics and find that related party transactions, negative media coverage, client concentration, internal audit, agency costs, equity pledges, equity incentives, employee quality and CEO behavioral integrity also affect audit fees [16–24]. Second, at the auditor level, studies have found that Big-N or industry specialist auditors usually charge higher audit fees because they are generally perceived to be of superior quality [25–28], and auditor tenure is also recognized as a factor affecting audit fees for the low-balling phenomenon [29]. Audit firm mergers increase the size and therefore also affect audit fees [30–32]. Some studies even extend to the individual auditor level, they argue that the personal characteristics of the auditor, such as gender, social relationships, and narcissism, etc. can also affect auditor fee [33–37]. However, it is difficult to measure variables in this area, therefore the research is relatively limited. Apart from that, some obvious audit engagement characteristic variables, such as audit opinion, audit report date, and non-audit services etc., are naturally considered into the audit fee model [34, 38, 39].

In summary, studies of international accounting network memberships have focused on the impact on audit quality, but some scholars argue that the inconsistency in the measurement of audit quality leads to less credibility of the final conclusion. In contrast, audit fee data are more objective and realistic to obtain. In terms of the current research on the factors influencing audit fees, the existing literature has paid little attention to the impact of international accounting networks’ specific characteristics on audit fees. Therefore, we can further focus on the impact of international accounting network memberships on audit fees in order to further explore the effect of audit firms’ internationalization.

### 2.2 Hypothesis development

Audit firm networks are hybrid governance structures between market and hierarchical organizations, formed by contractual relationships between legally and economically autonomous partnership entities from different countries. Network members can use the network brand, share resources (e.g., professionals, audit manuals, databases, and audit software), and make independent decisions in their respective markets; however, they are also subject to obligations such as considering global quality standards and quality reviews, submitting foreign audit work to network member firms in specific countries, and sharing network costs [40]. Existing studies have found that local firms joining international networks usually improve audit quality, but are the audit services provided by network members "good value for money" or "good quality for high price"? This needs to be explored in depth. Specifically, the impact of international accounting network memberships on audit fees can be analyzed from the following aspects.

From the perspective of reputation, joining an international network can improve auditor reputation, which in turn affects audit fees. Firms hiring auditors with higher reputation can alleviate information asymmetry, increase availability of bank loan [41], lower equity financing costs [42], reduce the IPO price suppression rate [43], and increase the value of insurance, so auditors with higher reputation can obtain a premium. By joining the international network, the audit firm can put the network brand on its official website to enhance the audit firm’s reputation and market recognition. In addition, although members of international networks are legally independent of each other and do not bear the legal risk for violations by other members, audit failures can still negatively affect the reputation of other members, which in turn affects the resignation of their audit clients. Therefore, the networks employ more stringent criteria for the selection of member firms, and once an audit firm enters the international network, it sends a reputational signal to the market that the member firm’s audit capability and professional ethics are at a high level. Therefore, member firms will charge more for the audit services due to their higher reputation.

In addition, by participating in network members’ meetings, using resources such as members-only Intranets, audit software and information sessions, members are able to improve professional competence and effectively detect misstatements, which in turn may result in higher audit fees. *“During the five years of cooperation with BDO from 2002 to 2007*, *our international business department has developed a group of talents and accumulated some experience*,*”* said Reanda’s Chief partner in an interview. The principal partner of Dahua, Chun Liang, also said that *through the cooperation with BDO*, *Dahua has improved its professional capacity and management experience and learned more international thinking and service consciousness*. These interview responses are consistent with the findings of a survey study by Bills et al., they found that small firms often use international network memberships to obtain necessary resources and improve their market position, and that most respondents believe that the resources provided by international networks, especially professional skills, are very important for audit quality [3]. Above evidence shows that joining an international firm can help members improve their professional skills, thereby increasing audit fees. Based on above analysis, this paper proposes the following hypothesis.

Hypothesis 1: Member firms will charge higher audit fees than non-members from their clients.

The specific characteristics of the international networks to which the firm belongs may have a further impact on audit fees. Audit firm size is often used as a proxy variable for reputation and professional competence [43], an international network with higher revenue or ranking will thus have more pronounced reputational and technical spillover effects on its members. Therefore, the higher the total revenue and the ranking of the international network the audit firm joins, the higher the audit fees are likely to be.

According to the Global Survey Report published by the International Accounting Bulletin, the international networks’ revenue consists of five main components, audit and assurance, accounting, tax, consulting and others. The revenue composition reflects, to some extent, the audit firm’s specialization in practice. The higher the proportion of audit revenue in the total revenue of an international network, the more the international network focuses on audit business development. Naturally, the member firms can also receive more professional skills training and knowledge sharing in the audit field, and then charge higher audit fees. Therefore, this paper proposes the following hypothesis.

Hypothesis 2: The revenue, ranking or audit engagement’ percentage of the international networks the audit firms have joined positively affect the audit fees the member firms may charge.

## 3. Research design

### 3.1 Sample selection and data source

This paper takes A-share listed companies from 2007–2019 as the research sample, and excludes: (1) financial listed companies; (2) ST and *ST listed companies; (3) companies with missing value. In addition, we exclude the sample with an interval of less than 1 year between the time of the audit firm’s new merger and the time of joining the international network and finally getting a total of 25,491 sample observations. Table 1 shows our processing steps. The data of audit firms joining international networks are obtained manually based on the information disclosed on the websites of the Chinese Institute of Certified Public Accountants (CICPA, http://cmis.cicpa.org.cn) and the official websites of each audit firm. The data of international networks’ revenue, ranking and the proportion of audit business are obtained from the 2011–2019 Global Survey Report published by the International Accounting Bulletin. All other data are collected from the CSMAR database. To avoid the effect of extreme values, all continuous variables in this paper have been winsorized by 1% at the top and bottom. This paper uses STATA 16.0 software to analyze and process the data.

**Table 1:** Sample selection.

Firm-year observations from 2007 to 2019	33922
Less: Non-A-share listed companies	(261)
Less: Financial listed companies	(2001)
Less: ST and *ST listed companies	(1797)
Less: Observations missing data to construct control variables	(3990)
Less: sample with an interval of less than 1 year between the time of the audit firm’s new merger and the time of joining the international network	(382)
Final sample of firm-years observations for analyses	25491

### 3.2 Variable definition and model construction

#### 3.2.1 Independent variable- member and characteristic

Drawing on the study of Mao et al. (2017) [2], *MEMBER* takes the value of 1 if the audit firm hired by the company in the current year is a member of an international accounting network, and 0 otherwise. It should be noted that in order to avoid the overlap effect generated by the audit firm merger event, we exclude the sample with an interval of less than 1 year between the time of the audit firm’s new merger and the time of joining the international network.

#### 3.2.2 Empirical model

The regression model of this paper is shown as follows.


$$FEE=\alpha_{0}+\alpha_{1}MEMBER+\alpha_{2}CONTROLS+\Sigma Industry+\Sigma Year+\varepsilon$$ (1)

$$FEE=\beta_{0}+\beta_{1}Characteristic+\beta_{2}CONTROLS+\Sigma Industry+\Sigma Year+\varepsilon$$ (2)

We test the effect of international network memberships on audit fees by model (1), *MEMBER* indicates whether they are members of international networks, *FEE* is the natural logarithm of audit fees, and the expected coefficient α_1_ is significantly positive. Model (2) tests the impact of international networks’ revenue, ranking, and audit and accounting practice share on audit fees, *Characteristic* means international network revenue (*REV*), ranking (*RANK*), and audit and accounting practice share (*AUD_SPE*), respectively, and the expected coefficient β1 is significantly positive. The control variables include basic company characteristics variables, corporate governance variables, and audit-related variables that may have an impact on audit fees, they are company size (*SIZE*), gearing ratio (*LEV*), current ratio (*CR*), business complexity (*INVE*, *REC*), return on net assets (*ROE*), loss or not (*LOSS*), total asset turnover ratio (*ATR*), and company growth (*GROWTH*), majority shareholder ownership (*TOP5*), number of subsidiaries (*SUBNUM*), issuance of new shares (*ISSUE*), auditor change (*SWITCH*), audit opinion (*OP*), and Big 4 or not (*BIG4*), and the model further controls for industry and year fixed effects. The specific variables are defined as shown in Table 2.

**Table 2:** Variables definitions.

Variables	Definition
*FEE*	Natural logarithm of audit fees;
*MEMBER*	Equal to 1 if the audit firm is a member of international networks, 0 otherwise;
*RANK*	The reverse of the ranking of international networks;
*REV*	Natural logarithm of international networks’ revenue;
*AUD_SPE* *	The ratio of audit and accounting practice revenue to total revenue of international networks;
*SIZE*	Company size, measured as the natural logarithm of total assets;
*LEV*	The ratio of total liabilities to total assets;
*CR*	The ratio of current liabilities to current assets;
*INVE*	The inventories scaled by total assets;
*REC*	The account receivables scaled by total assets;
*GROWTH*	Growth rate of main operating revenue = current year’ principal operation revenue / last year’ principal operation revenue -1;
*ROE*	Return on net assets, computed as the net profits scaled by net assets;
*LOSS*	An indicator variable for losses, equaling 1 if net profit less than zero, 0 otherwise;
*ATR*	The operating incomes scaled by total assets;
*TOP5*	The number of shares held by top five shareholders divided by total number of shares;
*SUBNUM*	Number of subsidiaries of the company;
*ISSUE*	An indicator variable for new shares issuing, equaling 1 if the company issues new shares in the current year and 0 otherwise;
*SWITCH*	An indicator variable for audit firm change, equaling 1 if the audit firm is different from last year and 0 otherwise;
*OP*	An indicator variable for audit opinion, equaling 1 if the audit opinion is standard unqualified and 0 otherwise;
*BIG4*	An indicator variable for the audit firm, equaling 1 if it is a Big Four and 0 otherwise.

*Global surveys published in 2011–2015 by the International Accounting Bulletin divided the international network revenue into seven segments: audit and accounting, tax practice, management consulting, corporate finance, corporate restructuring/insolvency, litigation support and other practices; while the surveys published in 2016–2019 divided them into five segments: audit and assurance, accounting, tax, consulting and other practices. To guarantee comparable data, this paper uses the sum of two segments—audit and assurance and accounting in 2016–2019 to measure audit and accounting proportion.

## 4. Empirical analysis

### 4.1 Descriptive statistics

Panel A in Table 3 shows the descriptive statistics of the full sample, it can be seen that 77.3% of the audit firms employed by the sample companies are members of international networks, reflecting that the current audit market is dominated by them. From the other control variables, it can be seen that 10.1% of the sample companies have a loss in the previous year, the average share of the top five shareholders is 52.92%, 11.8% of the sample companies issue new shares, 15.3% of the sample companies change auditors, and the percentage of sample companies audited by Big 4 firms is 6.1%. Panel B reflects the characteristics of international networks, and the samples are less because the data is obtained based on the global surveys published by the International Accounting Bulletin in 2011-2019.The results of Panel B data show that the international networks’ average share of revenue in audit and accounting is 53.5%, so non-assurance business still accounts for a large proportion.

**Table 3:** Descriptive statistics.

Panel A: Full sample						
Variable	N	Mean	SD	P25	P50	P75
*FEE*	25491	13.716	0.724	13.218	13.592	14.078
*MEMBER*	25491	0.773	0.419	1.000	1.000	1.000
*SIZE*	25491	22.141	1.301	21.199	21.960	22.878
*LEV*	25491	0.553	0.206	0.396	0.555	0.714
*CR*	25491	2.218	2.207	1.071	1.547	2.428
*INVE*	25491	0.156	0.146	0.061	0.119	0.198
*REC*	25491	0.114	0.104	0.028	0.089	0.171
*ROE*	25491	0.145	0.265	0.024	0.078	0.191
*LOSS*	25491	0.101	0.301	0.000	0.000	0.000
*ATR*	25491	0.669	0.477	0.356	0.557	0.836
*GROWTH*	25491	0.181	0.428	-0.021	0.110	0.277
*TOP5*	25491	52.919	15.376	41.531	53.049	64.283
*SUBNUM*	25491	17.564	22.733	5.000	10.000	20.000
*ISSUE*	25491	0.118	0.322	0.000	0.000	0.000
*SWITCH*	25491	0.153	0.360	0.000	0.000	0.000
*OP*	25491	0.967	0.179	1.000	1.000	1.000
*BIG4*	25491	0.061	0.239	0.000	0.000	0.000
Panel B: International network members						
Variable	N	Mean	SD	P25	P50	P75
*REV*	16411	8.179	1.076	7.724	8.291	8.760
*RANK*	16411	0.220	0.262	0.0830	0.143	0.200
*AUD_SPE*	16426	0.535	0.0980	0.480	0.510	0.590

### 4.2 Correlation analysis

Table 4 reports the Pearson correlation coefficients between the variables. The results show that there is a significant positive correlation between international memberships and audit fees without controlling for other variables, which initially verifies the hypothesis 1. From the results of other control variables, it is clear that companies with higher audit fees are characterized by larger size, less liquidity, lower profitability, higher growth, higher shareholding of major shareholders, and a larger number of subsidiaries. Audit fees are also higher for companies that do not change auditors, without standard unqualified audit opinions, and audited by the Big 4. In addition, the correlation coefficients of the variables are mostly less than 0.3, indicating that there is no significant multicollinearity.

**Table 4:** Correlation analysis.

	*FEE*	*MEMBER*	*SIZE*	*LEV*	*CR*	*INVE*	*REC*	*ROE*	*LOSS*	*ATR*	*GROWTH*	*TOP5*	*SUBNUM*	*ISSUE*	*SWITCH*	*OP*	*BIG4*
*FEE*	1																
*MEMBER*	0.233***	1															
*SIZE*	0.764***	0.146***	1														
*LEV*	-0.321***	0.026***	-0.479***	1													
*CR*	-0.263***	0.024***	-0.333***	0.644***	1												
*INVE*	0.00800	-0.042***	0.101***	-0.314***	-0.083***	1											
*REC*	-0.081***	0.075***	-0.191***	0.031***	0.023***	-0.104***	1										
*ROE*	-0.163***	-0.00100	-0.178***	0.559***	0.613***	-0.167***	-0.044***	1									
*LOSS*	0.00500	0.010*	-0.074***	-0.177***	-0.101***	-0.012*	-0.011*	-0.410***	1								
*ATR*	0.080***	-0.055***	0.038***	-0.127***	-0.155***	0.018***	0.134***	0.023***	-0.103***	1							
*GROWTH*	0.011*	0.00300	0.055***	-0.036***	-0.040***	0.029***	0.035***	0.098***	-0.181***	0.141***	1						
*TOP5*	0.177***	0.072***	0.187***	0.057***	0.072***	-0.036***	-0.035***	0.177***	-0.131***	0.070***	0.066***	1					
*SUBNUM*	0.532***	0.093***	0.520***	-0.274***	-0.174***	0.117***	-0.065***	-0.115***	-0.026***	0.068***	0.030***	0.021***	1				
*ISSUE*	0.00300	-0.00300	0.015**	-0.055***	-0.064***	-0.015**	0.050***	-0.021***	-0.025***	0.022***	0.094***	-0.00200	0.00300	1			
*SWITCH*	-0.042***	0.00700	-0.015**	-0.035***	0.00400	0.017***	0.00400	-0.018***	0.023***	0.011*	0	0.00400	-0.023***	-0.031***	1		
*OP*	-0.017***	-0.013**	0.064***	0.136***	0.062***	0.030***	-0.00100	0.178***	-0.287***	0.059***	0.074***	0.087***	0.00600	0.043***	-0.037***	1	
*BIG4*	0.464***	0.138***	0.358***	-0.105***	-0.096***	-0.019***	-0.082***	-0.019***	-0.036***	0.043***	-0.009	0.194***	0.142***	-0.017***	0.008	0.022***	1

Note

***, **, * indicate statistically significant at the 1%, 5%, and 10% levels, respectively.

### 4.3 Multiple regression analysis

The regression results of international audit network memberships and audit fees are shown in Table 5. The results show that international network memberships are significantly positively related to audit fees at the 1% level, which indicates that audit fees are higher when companies hire audit firms that are members of international networks, validating the inference of hypothesis 1. The results for control variables are also consistent with expectations, showing that audit fees are higher when companies are larger, less liquid or have higher business complexity. Conversely, audit fees are lower when auditors change or standard unqualified audit opinions are issued. In addition, Big 4 audit fees are significantly higher than non-Big 4 audit fees.

**Table 5:** International accounting network memberships and audit fees.

	(1)	(2)	(3)
*MEMBER*	0.092***	0.094***	0.039***
	(6.64)	(6.97)	(3.19)
*SIZE*	0.418***	0.367***	0.319***
	(45.36)	(36.15)	(39.74)
*LEV*	0.173***	0.167***	0.117***
	(3.41)	(3.36)	(2.68)
*CR*	-0.010***	-0.012***	-0.011***
	(-2.90)	(-3.84)	(-3.77)
*INVE*	-0.166***	-0.182***	-0.095*
	(-2.94)	(-3.34)	(-1.90)
*REC*	0.056	0.004	-0.001
	(0.80)	(0.07)	(-0.01)
*ROE*	-0.064**	-0.069***	-0.060***
	(-2.48)	(-2.76)	(-2.67)
*LOSS*	0.134***	0.126***	0.094***
	(9.35)	(9.13)	(7.60)
*ATR*	0.124***	0.108***	0.095***
	(6.84)	(5.99)	(6.20)
*GROWTH*		-0.039***	-0.021***
		(-4.77)	(-2.79)
*TOP5*		0.002***	0.001**
		(4.63)	(2.44)
*SUBNUM*		0.005***	0.005***
		(12.20)	(14.58)
*ISSUE*		-0.007	0.004
		(-0.79)	(0.50)
*SWITCH*			-0.036***
			(-4.67)
*OP*			-0.171***
			(-8.63)
*BIG4*			0.716***
			(18.52)
*CONSTANT*	4.050***	5.026***	6.284***
	(18.90)	(21.48)	(34.20)
*IND*	Yes	Yes	Yes
*YEAR*	Yes	Yes	Yes
*Observations*	25491	25491	25491
*Adjusted R* ^*2*^	0.649	0.668	0.715

T-statistics are reported in parentheses.

***, ** and * indicate statistically significant at the 1%, 5%, and 10% levels, respectively. The standard errors are clustered by firm level in the regressions, and the same is done in the following tables.

Table 6 reports the regression results between the specific characteristics of the international networks the audit firms have joined and the audit fees. The results in column (1) show that the coefficient on *REV* is significantly positive at the 10% statistical level. In addition, since international accounting networks and international accounting alliances are ranked separately, columns (2) and (3) report the effects of networks’ and alliances’ ranking on audit fees, respectively. Columns (2) show a significant and positive correlation at the 5% level between networks’ ranking and audit fees, but the positive effect of alliances’ ranking on audit fees is not significant, which may be a result of the small sample size. The results in column (4) show that the higher the proportion of audit and accounting practices, the higher the audit fees, and it is significant at the 1% level. The above results verify the inference of hypothesis 2, that is, when the audit firm joins an international network with larger total revenue, higher ranking, and with audit and accounting practices as the main business, the audit fees charged by the audit firm are higher.

**Table 6:** Characteristics of international networks and audit fees.

	(1)	(2)	(3)	(4)
*REV*	0.013*			
	(1.85)			
*RANK*		0.160**	0.037	
		(2.20)	(0.80)	
*AUD_SPE*				0.373***
				(6.04)
*SIZE*	0.328***	0.325***	0.283***	0.321***
	(37.31)	(34.58)	(13.95)	(36.22)
*LEV*	0.212***	0.102*	-0.229*	0.060
	(4.06)	(1.85)	(-1.94)	(1.16)
*CR*	-0.012***	-0.008**	0.001	-0.007**
	(-3.51)	(-2.37)	(0.07)	(-2.17)
*INVE*	-0.223***	-0.092	-0.363**	-0.135**
	(-4.31)	(-1.47)	(-2.55)	(-2.26)
*REC*	0.148**	0.046	-0.343**	0.011
	(2.36)	(0.65)	(-2.04)	(0.17)
*ROE*	-0.132***	-0.111***	-0.097	-0.107***
	(-4.90)	(-4.02)	(-1.11)	(-4.02)
*LOSS*	0.099***	0.089***	0.067	0.086***
	(6.49)	(5.48)	(1.59)	(5.67)
*ATR*	0.102***	0.093***	0.105**	0.095***
	(6.00)	(4.96)	(2.08)	(5.25)
*GROWTH*	-0.006	-0.011	0.015	-0.009
	(-0.61)	(-1.07)	(0.55)	(-0.90)
*TOP5*	0.001***	0.001***	0.002*	0.001***
	(3.12)	(2.64)	(1.77)	(2.95)
*SUBNUM*	0.006***	0.005***	0.006***	0.005***
	(14.79)	(12.06)	(7.22)	(13.63)
*ISSUE*	-0.016*	0.009	-0.025	0.007
	(-1.65)	(0.89)	(-0.78)	(0.69)
*SWITCH*	-0.081***	-0.051***	0.060**	-0.035***
	(-9.24)	(-4.78)	(2.13)	(-3.48)
*OP*	-0.218***	-0.169***	-0.215***	-0.171***
	(-8.65)	(-6.32)	(-4.13)	(-6.93)
*BIG4*	0.584***	0.569***	0.000	0.701***
	(14.28)	(11.96)	(.)	(18.09)
*CONSTANT*	6.295***	6.328***	7.148***	6.225***
	(29.31)	(28.96)	(15.40)	(29.90)
*IND*	Yes	Yes	Yes	Yes
*YEAR*	Yes	Yes	Yes	Yes
*Observations*	16411	14755	1656	16426
*Adjusted R* ^*2*^	0.688	0.710	0.686	0.707

T-statistics are reported in parentheses.

***, ** and * indicate statistically significant at the 1%, 5%, and 10% levels, respectively.

## 5. Further analysis

### 5.1 Cross-sectional analysis

Economic policy uncertainty has a significant impact on micro-firm behavior. Existing literatures suggest that economic policy uncertainty exposes firms to greater risk, raises the cost of corporate finance and reduces the availability of credit resources [44, 45]. Economic policy uncertainty increases information asymmetry between auditors and clients, which in turn increases audit risk and audit fees [46]. International network members usually have a higher reputation and therefore suffer more serious losses in the event of an audit failure. Thus, when companies face higher economic policy uncertainty, member audit firms will expend more effort and therefore may charge more premiums for cost and risk compensation.

Based on this, the sample is divided into two groups according to whether EPU is greater than the median to test the moderating effect of EPU on the positive relationship between international memberships and audit fees. Drawing on the study of Baker et al. (2016) [47], EPU is measured by the economic policy uncertainty index jointly published by Stanford University and the University of Chicago. The results in column (1) of Table 7 show that the coefficient of *MEMBER* is statistically significant at the 1% level, indicating that the positive relationship between international memberships and audit fees is more significant when EPU is higher.

**Table 7:** Cross-sectional analysis: Economic policy uncertainty.

	(1)	(2)
	Higher EPU	Lower EPU
*MEMBER*	0.060***	0.010
	(4.01)	(0.73)
*SIZE*	0.316***	0.320***
	(39.62)	(33.23)
*LEV*	0.108**	0.112**
	(2.30)	(2.19)
*CR*	-0.011***	-0.012***
	(-3.42)	(-3.12)
*INVE*	-0.135**	-0.069
	(-2.49)	(-1.19)
*REC*	0.020	-0.051
	(0.32)	(-0.71)
*ROE*	-0.078***	-0.035
	(-3.17)	(-1.16)
*LOSS*	0.095***	0.095***
	(6.19)	(6.05)
*ATR*	0.097***	0.091***
	(5.86)	(5.39)
*GROWTH*	-0.014	-0.028***
	(-1.49)	(-2.71)
*TOP5*	0.001***	0.001
	(3.32)	(1.26)
*SUBNUM*	0.005***	0.006***
	(14.36)	(11.61)
*ISSUE*	-0.001	0.009
	(-0.09)	(0.92)
*SWITCH*	-0.029***	-0.051***
	(-2.98)	(-4.08)
*OP*	-0.173***	-0.167***
	(-7.11)	(-5.98)
*BIG4*	0.633***	0.822***
	(17.29)	(17.42)
*CONSTANT*	6.433***	6.263***
	(34.12)	(28.82)
*IND*	Yes	Yes
*YEAR*	Yes	Yes
*Observations*	14606	10885
*Adjusted R* ^*2*^	0.696	0.721

T-statistics are reported in parentheses.

***, ** and * indicate statistically significant at the 1%, 5%, and 10% levels, respectively.

### 5.2 Intermediary effect test

By joining an international network, audit firms can participate in member meetings, use resources such as members-only intranet, audit software and information exchange sessions, thus improving the professional competence, and in particular, gaining experience and competence in handling international business and enhancing auditors’ overseas expertise, which can ultimately provide better services to audit clients and increase audit fees. To verify the inference, we include auditors’ overseas expertise to test the intermediary effect. Drawing on Gunn and Michas (2018) [48], the auditor’s level of overseas expertise (*MULTINAT_EXP1*) is measured by the mean value of the internationalization level of clients audited by the accounting firm. The specific calculation process is as follows: first, the internationalization level of a company is equal to the overseas operating revenue divided by the total operating revenue; second, the average value of the internationalization level of clients audited by the audit firm each year is calculated, which is the sum of the internationalization level of all clients audited by the auditor in that year divided by the number of clients audited. In addition, the sum of the number of overseas subsidiaries of the clients in the year divided by the number of clients audited is also used to measure the auditor’s overseas expertise (*MULTINAT_EXP2*). The following intermediary effect model is constructed for testing.


$$MULTINAT\_EXP=\gamma_{0}+\gamma_{1}MEMBER+\gamma_{2}CONTROLS+\Sigma Industry+\Sigma Year+\varepsilon$$ (3)

$$FEE=\lambda_{0}+\lambda_{1}MEMBER+\lambda_{2}MULTINAT\_EXP+\lambda_{3}CONTROLS+\Sigma Industry+\Sigma Year+\varepsilon$$ (4)

The regression results of model (3) and model (4) are presented in Table 8. Columns (1) and (3) show that the coefficient γ_1_ of model (3) is significantly positive at the 10% and 1% statistical levels, respectively, indicating that joining international accounting networks can significantly improve the level of audit firms’ overseas expertise. Columns (2) and (4) show that the coefficient λ_2_ of model (4) is significantly positive at the 1% level, indicating that audit firms with higher overseas expertise often charge higher audit fees. γ_1_ and λ_2_ are both significant, then the indirect effect is significant. In addition, columns (2) and (4) show that the coefficient λ_1_ of model (4) is significantly positive at the 1% statistical level, and γ_1_*λ_2_ and λ_1_ are both positive, which indicates member audit firms charge higher audit fees, and the audit firms’ overseas expertise have partial mediation effect on the relationship.

**Table 8:** Intermediary effect test: Audit firms’ overseas expertise.

	(1)	(2)	(3)	(4)
	*MULTINAT_EXP1*	*FEE*	*MULTINAT_EXP2*	*FEE*
*MEMBER*	0.003*	0.035***	0.100***	0.035***
	(1.76)	(2.83)	(10.61)	(2.83)
*MULTINAT_EXP1*		0.692***		
		(5.04)		
*MULTINAT_EXP2*				0.046*
				(1.89)
*SIZE*	-0.001*	0.313***	0.001	0.319***
	(-1.87)	(39.49)	(0.53)	(39.56)
*LEV*	0.004	0.099**	0.023	0.116***
	(1.16)	(2.35)	(1.33)	(2.62)
*CR*	-0.000	-0.011***	-0.000	-0.011***
	(-1.25)	(-3.88)	(-0.32)	(-3.76)
*INVE*	0.008*	-0.111**	-0.005	-0.095*
	(1.78)	(-2.27)	(-0.25)	(-1.91)
*REC*	0.001	-0.004	-0.017	-0.011
	(0.22)	(-0.06)	(-0.58)	(-0.18)
*ROE*	0.005**	-0.062***	0.004	-0.065***
	(2.41)	(-2.80)	(0.39)	(-2.78)
*LOSS*	-0.001	0.091***	-0.001	0.095***
	(-0.85)	(7.45)	(-0.16)	(7.54)
*ATR*	0.002	0.093***	0.001	0.096***
	(1.58)	(6.16)	(0.20)	(6.17)
*GROWTH*	-0.001*	-0.019***	0.000	-0.022***
	(-1.90)	(-2.61)	(0.15)	(-2.89)
*TOP5*	0.000**	0.001**	0.000	0.001***
	(2.41)	(2.08)	(1.21)	(2.62)
*SUBNUM*	-0.000	0.005***	0.001***	0.005***
	(-0.26)	(14.84)	(6.63)	(14.37)
*ISSUE*	0.002**	0.002	0.002	0.003
	(2.46)	(0.25)	(0.43)	(0.39)
*SWITCH*	-0.001*	-0.034***	0.009**	-0.036***
	(-1.68)	(-4.37)	(2.42)	(-4.56)
*OP*	-0.001	-0.166***	0.006	-0.172***
	(-0.33)	(-8.39)	(0.63)	(-8.50)
*BIG4*	-0.002	0.707***	0.588***	0.678***
	(-1.15)	(16.88)	(57.32)	(16.16)
*CONSTANT*	0.117***	6.341***	0.055	6.283***
	(9.04)	(34.77)	(0.89)	(33.97)
*IND*	Yes	Yes	Yes	Yes
*YEAR*	Yes	Yes	Yes	Yes
*Observations*	24904	24904	24758	24758
*Adjusted R* ^*2*^	0.125	0.698	0.739	0.712

T-statistics are reported in parentheses.

***, ** and * indicate statistically significant at the 1%, 5%, and 10% levels, respectively

### 5.3 Economic consequences test

#### 5.3.1 International network memberships and abnormal audit fees

Excessive abnormal audit fees increase the auditor’s financial dependence on the client, which will have a negative impact on the quality of financial reporting [49, 50], and Hribar et al.(2014) also argue that abnormal audit fees are negatively related to audit quality, so abnormal audit fees often imply an audit collusion [51]. The previous analysis of this paper suggests that international network memberships will increase audit fees, so are the increased audit fees normal compensation for additional audit inputs or do they stem from an increase in abnormal audit fees with a hidden collusive motive? This paper argues that after joining an international accounting network, the audit firm’s professional competence and social reputation will be improved, so the audit fee is more a reward for the audit firm’s professional competence and diligent work rather than a risk compensation for audit collusion. Therefore, we expect that although international network memberships will increase total audit fees, the same will not happen to the abnormal audit fees.

To verify the above inference, drawing on the related research [52], abnormal audit fees (*AB_FEE1*) is measured as the rate of change in audit fees per unit of asset. On this basis, a dummy variable for abnormal audit fees (*AB_FEE2*) is constructed as whether abnormal audit fees (*AB_FEE1*) are greater than the annual average. The results are shown in columns (1) and (2) of Table 9, where international network memberships are significantly negatively related to abnormal audit fees at the 10% level, indicating that member audit firms reduce abnormal audit fees.

**Table 9:** Economic consequences test: Abnormal audit fees and financial reporting quality.

	(1)	(2)	(3)	(4)
	*AB_FEE1*	*AB_FEE2*	*ABS_DA1*	*ABS_DA2*
*MEMBER*	-0.006*	-0.067*	-0.003**	-0.003*
	(-1.68)	(-1.83)	(-2.25)	(-1.85)
*SIZE*	-0.016***	-0.146***	-0.004***	-0.005***
	(-8.67)	(-8.13)	(-6.56)	(-6.45)
*LEV*	0.051***	0.577***	-0.026***	-0.023***
	(4.01)	(4.95)	(-6.03)	(-4.83)
*CR*	0.004***	0.059***	0.002***	0.001**
	(3.23)	(5.97)	(4.12)	(1.97)
*INVE*	0.001	-0.140	0.039***	0.045***
	(0.07)	(-0.98)	(6.66)	(6.26)
*REC*	0.017	-0.059	0.010	0.008
	(1.05)	(-0.35)	(1.54)	(1.10)
*ROE*	-0.101***	-1.140***	-0.008**	0.000
	(-10.75)	(-12.48)	(-2.46)	(0.07)
*LOSS*	0.104***	0.935***	0.027***	0.027***
	(15.81)	(16.44)	(14.63)	(12.71)
*ATR*	-0.001	-0.020	0.003**	0.003*
	(-0.33)	(-0.52)	(2.28)	(1.69)
*GROWTH*	-0.105***	-0.625***	0.020***	0.022***
	(-20.41)	(-12.41)	(12.02)	(11.41)
*TOP5*	-0.001***	-0.005***	0.000	0.000
	(-6.88)	(-4.58)	(0.99)	(0.83)
*SUBNUM*	0.000***	0.002***	-0.000***	-0.000***
	(4.59)	(2.70)	(-3.27)	(-2.66)
*ISSUE*	-0.037***	-0.320***	0.004***	0.004**
	(-7.41)	(-6.72)	(2.84)	(2.49)
*SWITCH*	-0.004	-0.027	0.004***	0.003*
	(-0.86)	(-0.62)	(2.89)	(1.69)
*OP*	-0.123***	-0.571***	-0.034***	-0.033***
	(-10.30)	(-6.48)	(-8.72)	(-7.57)
*BIG4*	0.050***	0.188***	0.001	-0.002
	(6.98)	(2.58)	(0.29)	(-0.73)
*CONSTANT*	0.431***	1.714***	0.183***	0.193***
	(9.37)	(3.95)	(11.42)	(10.48)
*IND*	Yes	Yes	Yes	Yes
*YEAR*	Yes	Yes	Yes	Yes
*Observations*	24374	25491	25030	20103
*Adjusted R* ^*2*^	0.129		0.102	0.101
*Pseudo R* ^*2*^		0.109		

T-statistics are reported in parentheses.

***, ** and * indicate statistically significant at the 1%, 5%, and 10% levels, respectively.

#### 5.3.2 International network memberships and financial reporting quality

The previous results show that international memberships are significantly and negatively associated with abnormal audit fees, suggesting to some extent that member audit firms are less likely to engage in audit collusion in order to maintain their good reputation. To further verify the inference, it is necessary to test the relationship between international memberships and financial reporting quality. Drawing on the studies of Kothari et al. (2005) and Dechow et al. (2003) [53, 54], the absolute value of discretional accruals, ABS_DA1 and ABS_DA2, are calculated to measure the quality of financial reporting, respectively.

Column 3 of Table 8 represents the results out of the Kothari et al. (2005) model and column 4 represents the results out of the Dechow (2003) model. The coefficient of MEMBER is significantly negative at the 5% and 10% statistical level, respectively, indicating that international memberships can significantly reduce the degree of audit clients’ earnings management and improve the quality of financial reporting. The result also verifies that higher audit fees are more likely to be additional compensation for auditors’ hard work to improve financial reporting quality.

## 6. Robustness tests

### 6.1 Endogeneity test

#### 6.1.1 Endogeneity test of hypothesis 1

First, considering the influence of individual company characteristics on the results, we regress by a fixed-effects model, and the results are shown in column (1) of Table 10, where the coefficient of *MEMBER* is still significantly positive.

**Table 10:** Endogeneity test of hypothesis 1.

	(1)	(2)	(3)
	fixed-effects model	DID	the second stage of Heckman two-stage model
*MEMBER*	0.019*		0.036**
	(1.95)		(2.20)
*MEMBERPOST*		0.034***	
		(2.68)	
*SIZE*	0.307***	0.311***	0.325***
	(27.76)	(25.65)	(38.27)
*LEV*	0.024	0.019	0.146***
	(0.65)	(0.48)	(3.11)
*CR*	-0.008***	-0.007***	-0.013***
	(-3.52)	(-3.01)	(-3.93)
*INVE*	-0.092*	-0.113**	-0.089*
	(-1.86)	(-2.03)	(-1.68)
*REC*	0.083	0.068	-0.011
	(1.23)	(0.93)	(-0.16)
*ROE*	-0.003	-0.006	-0.087***
	(-0.17)	(-0.33)	(-3.46)
*LOSS*	0.045***	0.046***	0.092***
	(6.23)	(5.92)	(7.01)
*ATR*	0.048***	0.059***	0.098***
	(2.76)	(3.06)	(5.87)
*GROWTH*	-0.014**	-0.016**	-0.017**
	(-2.31)	(-2.49)	(-2.00)
*TOP5*	0.001	0.000	0.001*
	(1.37)	(0.94)	(1.78)
*SUBNUM*	0.003***	0.003***	0.005***
	(9.10)	(8.34)	(14.11)
*ISSUE*	-0.003	-0.002	0.004
	(-0.48)	(-0.43)	(0.49)
*SWITCH*	-0.009*	-0.012**	-0.032***
	(-1.77)	(-2.11)	(-3.76)
*OP*	-0.120***	-0.123***	-0.159***
	(-8.33)	(-7.78)	(-7.71)
*BIG4*	0.322***	0.301***	0.695***
	(7.45)	(6.38)	(17.58)
*CONSTANT*	6.560***	6.501***	6.573***
	(26.91)	(24.27)	(32.45)
*IND*	Yes	Yes	Yes
*YEAR*	Yes	Yes	Yes
*Observations*	25491	22526	21356
*Adjusted R* ^*2*^	0.701	0.698	0.710

T-statistics are reported in parentheses.

***, ** and * indicate statistically significant at the 1%, 5%, and 10% levels, respectively.

Second, we use difference-in-difference method to alleviate the problem of self-selection. Specifically, the model treats the audit firm weather chooses to join an international network as a policy implementation, and the sample that changes from a member firm to a non-member firm during the sample period is excluded. Given the inconsistency in the timing of each audit firm becoming an international network member, the asymptotic difference-in-difference model is constructed as follows.


$$FEE=\alpha_{0}+\alpha_{1}MEMBERPOST+\alpha_{2}CONTROLS+\Sigma FIRM+\Sigma Year+\Sigma Industry+\varepsilon$$ (5)

Where *MEMBERPOST* refers to whether the audit firm selected by the company is a member of an international network and the time is in the period after the audit firm becoming an international member, and the model controls for individual firm dummy variables and annual dummy variables. The regression results, as shown in column (2) of Table 10, indicate that audit fees increase significantly after an audit firm chooses to join an international network, validating the findings of this paper.

Finally, to avoid the endogeneity problem caused by reverse causality, this paper regresses with Heckman two-stage model by using whether the audit firm is a member firm in the previous year as the instrumental variable. The regression results of the second stage are shown in column (3) of Table 10, where the conclusion remains robust.

#### 6.1.2 Endogeneity test of hypothesis 2

For hypothesis 2, the fixed-effects model and the Heckman two-stage model are also used for endogeneity test. The regression results of the fixed-effects model are shown in columns (1)-(3) of Table 11, and the results remain robust except for column (1). In addition, the endogeneity test is also performed by using the Heckman two-stage model with lags of international networks’ revenue, ranking, and accounting and auditing practice share as instrumental variables, and the regression results of the second stage are shown in columns (4)-(6) of Table 11, and the results are still robust.

**Table 11:** Endogeneity test of hypothesis 2.

	fixed-effects model			the second stage of Heckman two-stage model		
	(1)	(2)	(3)	(4)	(5)	(6)
*REV*	0.002			0.024***		
	(0.32)			(2.80)		
*RANK*		0.079*			0.356***	
		(1.68)			(2.75)	
*AUD_SPE*			0.150**			0.412***
			(2.25)			(5.25)
*SIZE*	0.326***	0.332***	0.326***	0.326***	0.332***	0.327***
	(20.58)	(19.34)	(20.67)	(34.19)	(32.73)	(34.31)
*LEV*	0.005	0.022	0.003	0.072	0.103*	0.062
	(0.11)	(0.45)	(0.06)	(1.24)	(1.69)	(1.07)
*CR*	-0.006*	-0.005*	-0.005*	-0.008**	-0.009**	-0.008**
	(-1.95)	(-1.77)	(-1.91)	(-2.16)	(-2.30)	(-2.03)
*INVE*	-0.057	-0.028	-0.057	-0.127*	-0.091	-0.142**
	(-0.90)	(-0.44)	(-0.90)	(-1.91)	(-1.32)	(-2.13)
*REC*	0.145*	0.160*	0.140*	0.003	0.038	0.012
	(1.77)	(1.81)	(1.71)	(0.03)	(0.48)	(0.16)
*ROE*	-0.022	-0.024	-0.022	-0.135***	-0.134***	-0.135***
	(-1.12)	(-1.16)	(-1.12)	(-4.51)	(-4.33)	(-4.48)
*LOSS*	0.041***	0.039***	0.041***	0.087***	0.093***	0.087***
	(5.13)	(4.59)	(5.12)	(5.39)	(5.45)	(5.38)
*ATR*	0.059**	0.071***	0.058**	0.092***	0.091***	0.092***
	(2.42)	(2.88)	(2.37)	(4.60)	(4.40)	(4.62)
*GROWTH*	-0.018**	-0.020***	-0.018**	-0.006	-0.012	-0.008
	(-2.57)	(-2.64)	(-2.57)	(-0.59)	(-1.02)	(-0.79)
*TOP5*	-0.000	-0.000	-0.000	0.001**	0.001**	0.001**
	(-0.37)	(-0.53)	(-0.34)	(2.44)	(2.18)	(2.50)
*SUBNUM*	0.002***	0.002***	0.003***	0.005***	0.005***	0.005***
	(7.16)	(6.46)	(7.24)	(12.46)	(11.30)	(12.83)
*ISSUE*	-0.010	-0.005	-0.009	0.016	0.017	0.016
	(-1.43)	(-0.72)	(-1.42)	(1.37)	(1.42)	(1.43)
*SWITCH*	-0.007	-0.013*	-0.007	-0.031***	-0.034***	-0.023**
	(-1.12)	(-1.86)	(-1.13)	(-2.68)	(-2.73)	(-2.02)
*OP*	-0.109***	-0.113***	-0.110***	-0.163***	-0.157***	-0.154***
	(-6.26)	(-5.74)	(-6.25)	(-6.09)	(-5.44)	(-5.79)
*BIG4*	0.272***	0.234***	0.300***	0.581***	0.472***	0.700***
	(5.09)	(3.79)	(5.75)	(13.56)	(7.37)	(17.44)
*CONSTANT*	6.309***	6.187***	6.263***	6.461***	6.517***	6.419***
	(17.24)	(15.98)	(17.26)	(27.02)	(27.20)	(28.04)
*IND*	Yes	Yes	Yes	Yes	Yes	Yes
*YEAR*	Yes	Yes	Yes	Yes	Yes	Yes
*Observations*	16411	14755	16426	12908	11706	12921
*Adjusted R* ^*2*^	0.586	0.590	0.587	0.702	0.707	0.704

T-statistics are reported in parentheses.

***, ** and * indicate statistically significant at the 1%, 5%, and 10% levels, respectively.

### 6.2 Change the measurement of the independent variable

#### 6.2.1 Change the measurement of international memberships

Since there are differences in the specific month of audit firms joining international networks, some firms join in July and later in the year, so there may be a lag in the impact brought by their memberships. To mitigate the impact, this paper determines that audit firms joining international networks in July and later are not recognized as members of international networks in this year, that is to say, the *MEMBER* variable for these samples takes the value of 0. The regression results of hypothesis 1 and hypothesis 2 are shown in Table 12, and the results remain robust.

**Table 12:** Change the measurement of international memberships.

	(1)	(2)	(3)	(4)
*MEMBER2*	0.035***			
	(2.95)			
*REV*		0.012*		
		(1.71)		
*RANK*			0.159**	
			(2.18)	
*AUD_SPE*				0.382***
				(6.14)
*SIZE*	0.319***	0.321***	0.326***	0.321***
	(39.72)	(35.46)	(33.93)	(35.61)
*LEV*	0.117***	0.061	0.092	0.049
	(2.69)	(1.15)	(1.64)	(0.94)
*CR*	-0.011***	-0.007**	-0.008**	-0.007**
	(-3.77)	(-2.08)	(-2.21)	(-2.00)
*INVE*	-0.095*	-0.119*	-0.086	-0.130**
	(-1.90)	(-1.96)	(-1.35)	(-2.15)
*REC*	-0.001	0.018	0.061	0.024
	(-0.02)	(0.26)	(0.85)	(0.35)
*ROE*	-0.060***	-0.110***	-0.112***	-0.109***
	(-2.68)	(-4.08)	(-4.06)	(-4.05)
*LOSS*	0.094***	0.085***	0.088***	0.085***
	(7.59)	(5.55)	(5.39)	(5.56)
*ATR*	0.095***	0.093***	0.091***	0.092***
	(6.21)	(4.98)	(4.67)	(4.97)
*GROWTH*	-0.021***	-0.008	-0.012	-0.009
	(-2.75)	(-0.82)	(-1.16)	(-0.97)
*TOP5*	0.001**	0.001***	0.001***	0.001***
	(2.44)	(3.09)	(2.79)	(3.09)
*SUBNUM*	0.005***	0.005***	0.005***	0.005***
	(14.59)	(13.38)	(12.11)	(13.71)
*ISSUE*	0.004	0.007	0.010	0.007
	(0.48)	(0.69)	(0.92)	(0.70)
*SWITCH*	-0.036***	-0.043***	-0.053***	-0.035***
	(-4.66)	(-4.25)	(-4.87)	(-3.48)
*OP*	-0.171***	-0.181***	-0.173***	-0.173***
	(-8.63)	(-7.23)	(-6.39)	(-6.97)
*BIG4*	0.716***	0.616***	0.567***	0.700***
	(18.52)	(15.15)	(11.92)	(18.02)
*CONSTANT*	6.284***	6.348***	6.325***	6.219***
	(34.20)	(29.14)	(28.46)	(29.43)
*IND*	Yes	Yes	Yes	Yes
*YEAR*	Yes	Yes	Yes	Yes
*Observations*	25491	15937	14281	15952
*Adjusted R* ^*2*^	0.715	0.707	0.712	0.709

T-statistics are reported in parentheses.

***, ** and * indicate statistically significant at the 1%, 5%, and 10% levels, respectively.

#### 6.2.2 Change the measurement of international networks’ characteristics

First, analogous to the unique reputation and brand effect of the international Big Four, this paper considers that the top 10 international accounting networks in the world also have unique brand effect, so the top 10 international networks are assigned a value of 1, otherwise they are assigned a value of 0. Second, dummy variables for international networks’ revenue, audit and accounting practice share are constructed, taking 1 if greater than the median value of current year and 0 otherwise. Regression results are shown in Table 13, and the conclusions remain the same.

**Table 13:** Change the measurement of international network’s characteristics.

	(1)	(2)	(3)
*REV2*	0.043***		
	(3.58)		
*RANK2*		0.036***	
		(2.75)	
*AUD_SPE2*			0.030***
			(2.86)
*SIZE*	0.320***	0.324***	0.321***
	(35.98)	(34.65)	(36.14)
*LEV*	0.070	0.104*	0.068
	(1.34)	(1.89)	(1.30)
*CR*	-0.008**	-0.009**	-0.008**
	(-2.26)	(-2.46)	(-2.27)
*INVE*	-0.119**	-0.091	-0.129**
	(-1.99)	(-1.46)	(-2.17)
*REC*	0.001	0.044	0.008
	(0.02)	(0.62)	(0.12)
*ROE*	-0.109***	-0.110***	-0.106***
	(-4.08)	(-4.00)	(-3.97)
*LOSS*	0.086***	0.089***	0.086***
	(5.71)	(5.52)	(5.68)
*ATR*	0.096***	0.093***	0.096***
	(5.24)	(4.95)	(5.29)
*GROWTH*	-0.007	-0.010	-0.008
	(-0.69)	(-1.01)	(-0.83)
*TOP5*	0.001***	0.001***	0.001***
	(2.89)	(2.64)	(2.97)
*SUBNUM*	0.005***	0.005***	0.005***
	(13.38)	(12.13)	(13.42)
*ISSUE*	0.007	0.009	0.007
	(0.66)	(0.83)	(0.71)
*SWITCH*	-0.043***	-0.055***	-0.040***
	(-4.34)	(-5.18)	(-4.02)
*OP*	-0.178***	-0.171***	-0.175***
	(-7.18)	(-6.40)	(-7.08)
*BIG4*	0.627***	0.626***	0.664***
	(16.66)	(16.47)	(17.54)
*CONSTANT*	6.441***	6.350***	6.412***
	(31.15)	(29.17)	(31.04)
*IND*	Yes	Yes	Yes
*YEAR*	Yes	Yes	Yes
*Observations*	16411	14770	16426
*Adjusted R* ^*2*^	0.706	0.710	0.706

T-statistics are reported in parentheses.

***, ** and * indicate statistically significant at the 1%, 5%, and 10% levels, respectively.

### 6.3 Change the scope of the sample

There are differences in the way Big Four audit firms cooperate with local audit firms compared to other international networks, and existing researches also distinguish between Big Four and non-Big Four international firms in their studies. Therefore, this paper excludes the sample audited by the international Big Four and regresses the results, which are still robust as shown in Table 14.

**Table 14:** Change the scope of the sample.

	(1)	(2)	(3)	(5)
*MEMBER*	0.028**			
	(2.27)			
*REV*		0.014**		
		(2.04)		
*RANK*			0.628***	
			(5.10)	
*AUD_SPE*				0.312***
				(4.98)
*SIZE*	0.296***	0.295***	0.295***	0.295***
	(36.25)	(31.84)	(30.25)	(31.98)
*LEV*	0.086**	0.037	0.071	0.028
	(2.08)	(0.73)	(1.33)	(0.56)
*CR*	-0.014***	-0.010***	-0.011***	-0.010***
	(-4.92)	(-3.03)	(-3.37)	(-2.96)
*INVE*	-0.115**	-0.143**	-0.105*	-0.154***
	(-2.36)	(-2.44)	(-1.71)	(-2.62)
*REC*	-0.023	-0.006	0.033	0.002
	(-0.38)	(-0.09)	(0.47)	(0.03)
*ROE*	-0.040*	-0.092***	-0.095***	-0.091***
	(-1.79)	(-3.45)	(-3.51)	(-3.43)
*LOSS*	0.086***	0.078***	0.080***	0.078***
	(7.03)	(5.24)	(4.99)	(5.27)
*ATR*	0.089***	0.084***	0.080***	0.083***
	(5.83)	(4.60)	(4.27)	(4.59)
*GROWTH*	-0.021***	-0.004	-0.007	-0.006
	(-2.75)	(-0.45)	(-0.69)	(-0.58)
*TOP5*	0.001*	0.001**	0.001**	0.001**
	(1.80)	(2.54)	(2.09)	(2.54)
*SUBNUM*	0.006***	0.006***	0.006***	0.006***
	(15.44)	(14.01)	(12.67)	(14.28)
*ISSUE*	-0.001	0.002	0.004	0.002
	(-0.08)	(0.23)	(0.39)	(0.24)
*SWITCH*	-0.022***	-0.027***	-0.038***	-0.021**
	(-2.81)	(-2.67)	(-3.49)	(-2.07)
*OP*	-0.160***	-0.162***	-0.154***	-0.155***
	(-8.14)	(-6.51)	(-5.74)	(-6.27)
*CONSTANT*	6.770***	6.893***	6.921***	6.819***
	(36.25)	(30.97)	(30.52)	(31.68)
*IND*	Yes	Yes	Yes	Yes
*YEAR*	Yes	Yes	Yes	Yes
*Observations*	23943	15178	13522	15193
*Adjusted R* ^*2*^	0.644	0.619	0.617	0.621

T-statistics are reported in parentheses.

***, ** and * indicate statistically significant at the 1%, 5%, and 10% levels, respectively.

## 7. Conclusions

This paper explores the intrinsic link between audit firms’ international network memberships and audit fees. It is found that, firstly, audit firms that join international networks charge significantly higher audit fees compared to non-members of international networks. Secondly, audit fees are higher if the international networks they join have higher revenues, higher rankings, or a larger share of audit and accounting practices. Third, further tests conclude that the positive effect of international network membership on audit fees is more significant when economic policy uncertainty is higher; mediating effects tests find that international network memberships affect audit fees through the path of increasing the level of overseas expertise. In addition, international network members are significantly negatively correlated with abnormal audit fees, and can improve the quality of financial reports. Our main results are consistent with Bills et al. (2016), suggesting that the findings might be transferable to other audit markets to some extent. Additionally, our paper investigates the relationship between specific characteristics of member firms and audit fees, and attempts to explore the rationalization of higher audit fees charged by member firms. The exploration of these delicate aspects can deepen the literatures about the relationship between member firms and audit fees.

This study provides the following insights. First, audit firms in China can enhance their reputation and expertise by joining international networks, and then improve their market competitiveness. Second, when choosing an audit firm, companies should consider whether the firm is an international network member and the specific characteristics of the international network it joins, so that they can choose a firm that better meets their needs. Third, government departments should provide support for the international development of audit firms, strengthen the rational allocation of resources in the audit market, and escort the CPA profession to become bigger and stronger.
